# The Impact of COVID-19 on Domestic Tourism by Older People in Taiwan

**DOI:** 10.3389/fpubh.2022.885632

**Published:** 2022-06-28

**Authors:** Ching-Tang Chan

**Affiliations:** Institute of National Development, National Taiwan University, Taipei, Taiwan

**Keywords:** tourism, COVID-19, Taiwan, healthy, older people

## Abstract

Taiwan's older population (those over the age of 65) reached ~3.95 million at the end of January 2022, accounting for around 16.9% of the country's total population. It is already an “aged society.” With the gradual increase in the older population, the older people tourism market is also getting more and more attention. This article explores how older people tourism was affected by the COVID-19 pandemic (present in Tawian from early 2020), which was a major international public health event. This study adopts quantitative and PCA methods to statistically analyze the changes before and after the pandemic. The study results found that the frequency of tourism decreased after the pandemic: the number was 5.32, a decrease of 0.77, and instances of at least 1 tourist trip decreased by 3.87% after the pandemic. Regarding the reasons for not participating in tourism, during the COVID-19 pandemic, the COVID-19 accounted for a factor of 19.9%. Total travel expenses were NT$2,590, an increase of NT$229.67, and were not affected by the pandemic. We carried out a PCA analysis on tourism spending. The first component was food, accommodation, shopping, and other expenses. The factor loadings were 0.989, 0.931, 0.641 and −0.948, respectively. The second component was entertainment and transportation expenses. The factor loadings were 0.997 and 0.902, respectively. In conclusion, we put forward relevant discussions and suggestions to make tourism for older people healthier and more sustainable.

## Introduction

The aging of the population is a common phenomenon all over the world (1). According to the definition of the World Health Organization, when the population of those aged 65 or older in a country accounts for more than 7% of the total population, it is called an “aging society,” when it is more than 14% it is called an “aged society,” and when it is more than 21% it is called a “super-aged society.”

Owing to the decline in the fertility rate and the increase in life expectancy, the young population continues to decrease and the older population to increase. Taiwan became an aging society in 1993 and an aged society in 2018 (2). Its population of those aged over 65 had reached 3.95 million by the end of January 2022 (3). It accounts for ~16.9% of the total population, and the country is gradually moving toward becoming a super-aged society.

At present, a major international public health event is generally declared by the Emergency Committee under the World Health Organization (WHO), as a “Public Health Emergency of International Concern” (PHEIC). According to the International Health Regulations established in 2005, this is defined as an extraordinary event constituting a public health risk to other states through the international spread of disease, and potentially requires a coordinated international response. The event is “serious, sudden, unusual or unexpected,” “carries implications for public health beyond the affected State's national border,” and “may require immediate international action” (4). COVID-19 was declared by the WHO emergency committee a Public Health Emergency of International Concern on 30 January 2020 (5).

The COVID-19 pandemic appeared in Taiwan in early 2020 (6), according to the database of the WHO and the Centers for Disease Control in Taiwan (7, 8), and the confirmed rates of infection and death in Taiwan are lower than the global average (Table 1). However, due to the emergence of successive mutant strains of COVID-19, the pandemic situation abroad is still severe, and most countries still have measures such as checks and quarantines in place on entry or exit. Therefore, the global tourism industry is still significantly affected by COVID-19.

**Table 1:** The cases of COVID-19 in Taiwan and worldwide in 2020 and 2021.

	**World cumulative case**		**World population** **(per million)**		**Taiwan cumulative case**		**Taiwan population** **(per million)**	
**Year**	**Confirmed case**	**Deaths**	**Confirmed rate**	**Death rate**	**Confirmed case**	**Deaths**	**Confirmed rate**	**Death rate**
2020	82,386,776	1,801,095	10,653.92,163	232.9,102,548	800	7	34.7,826,087	740.3913043
2021	285,626,807	5,428,585	36,293.11,398	689.7,820,839	17,029	850	34.7,826,087	36.95652174

*1. Confirmed rate = Confirmed case / Population*.

*2. Death rate = Deaths / Population*.

However, though the currently available literature discusses the impact of COVID-19 on tourism (9, 10), tourists, and travel destinations (11–14), there is still a lack of literature on its impact on tourism by the older people. Therefore, this paper analyses the changes this major international public health event has caused in older people tourism, and puts forward relevant discussions and suggestions to make tourism for older people healthier and more sustainable.

## Literature Review

There are some relevant articles describing the psychological distress and fear of COVID-19 experienced by older people during the COVID-19 pandemic. There was a study assessing the fear of COVID-19 among the older people in Iran and Taiwan (15). Although Iranian older people had a significantly higher level of fear of COVID-19 than Taiwanese older people, the latter still had a considerable fear of the disease. There was also a study to investigate the association between the fear of COVID-19 and preventive behaviors during the COVID-19 community outbreak of two severity levels in Taiwanese older people (16). The severity of a COVID-19 outbreak may alter older people's psychological status and related behaviors. Another study explored the gender differences related to the fear of COVID-19 among older women and men in Taiwan (17). As behaviors designed to prevent COVID-19 infection were associated with a lower fear of COVID-19, healthcare providers should consider strategies for improving preventive behaviors among the older people to help ease their worries and fears concerning COVID-19. Several studies have shown that during the pandemic, the older people, especially those with chronic diseases, were more vulnerable than youths (18, 19). This literature found older people's psychological distress, fear, and related behaviors were affected by the COVID-19 pandemic. It could explain why the number of older people tourism decreased during the COVID-19 pandemic.

## Materials and Methods

### Data Sources

This research uses the database of Taiwanese tourism of the Tourism Bureau of the Ministry of Transport (20), the COVID-19 database of the Ministry of Health and Human Services, the WHO COVID-19 database, and relevant information from the government's official website and reports.

### Research Methods

This study adopts quantitative methods to statistically analyze older people tourism in Taiwan from 2017 to 2020. We extracted the tourism-related data on the older people within the scope of the study from the above databases and information, and compiled it in Excel and SPSS 25 for consolidated analysis and illustrations to produce the tables and figures included. As a result of investigating the differences from before and after the pandemic, we also did comparative and trend analyses, which more clearly showed the impact of COVID-19.

After the outbreak of the pandemic, because of the entry-exit control and isolation measures, people mainly traveled domestically. Therefore, this paper focuses on the domestic travel of the older people in Taiwan. The study period was from 3 years before the outbreak, 2017–2019, to 1 year after the outbreak, 2020. The database of Taiwanese tourism status of the Tourism Bureau is a nationwide survey. The information needs to spend more time to investigate and update, and the latest information is from 2020.

Principal component analysis (PCA) was invented in 1901 by Karl Pearson, as an analog of the principal axis theorem in mechanics; it was later independently developed and named by Harold Hotelling in the 1930's (21, 22). PCA is a technique for reducing the dimensionality of large datasets, increasing interpretability, and at the same time minimizing information loss. It does so by creating new uncorrelated variables that successively maximize variance, hence making PCA an adaptive data analysis technique (23). PCA is a multivariate technique that analyzes a set of data in which observations can be described by several correlated variables (24, 25). PCA defines a new orthogonal coordinate system that optimally describes variance in a dataset (26). Varimax rotation is done in PCA, so that the first axis contains as much variation as possible, and the second axis contains as much of the remaining variation as possible. It maximizes the sum of the variances of the squared loadings as all the coefficients will be either large or near zero; the goal is to associate each variable to at most one factor (27). The amount spent by older people tourists contained many items; to find the main component that contributed to the tourism industry, this study applied the PCA analysis method to reduce the number of variables and further understand the relationships between the items.

## Results

### Frequency of Tourism

**Figure 1:**
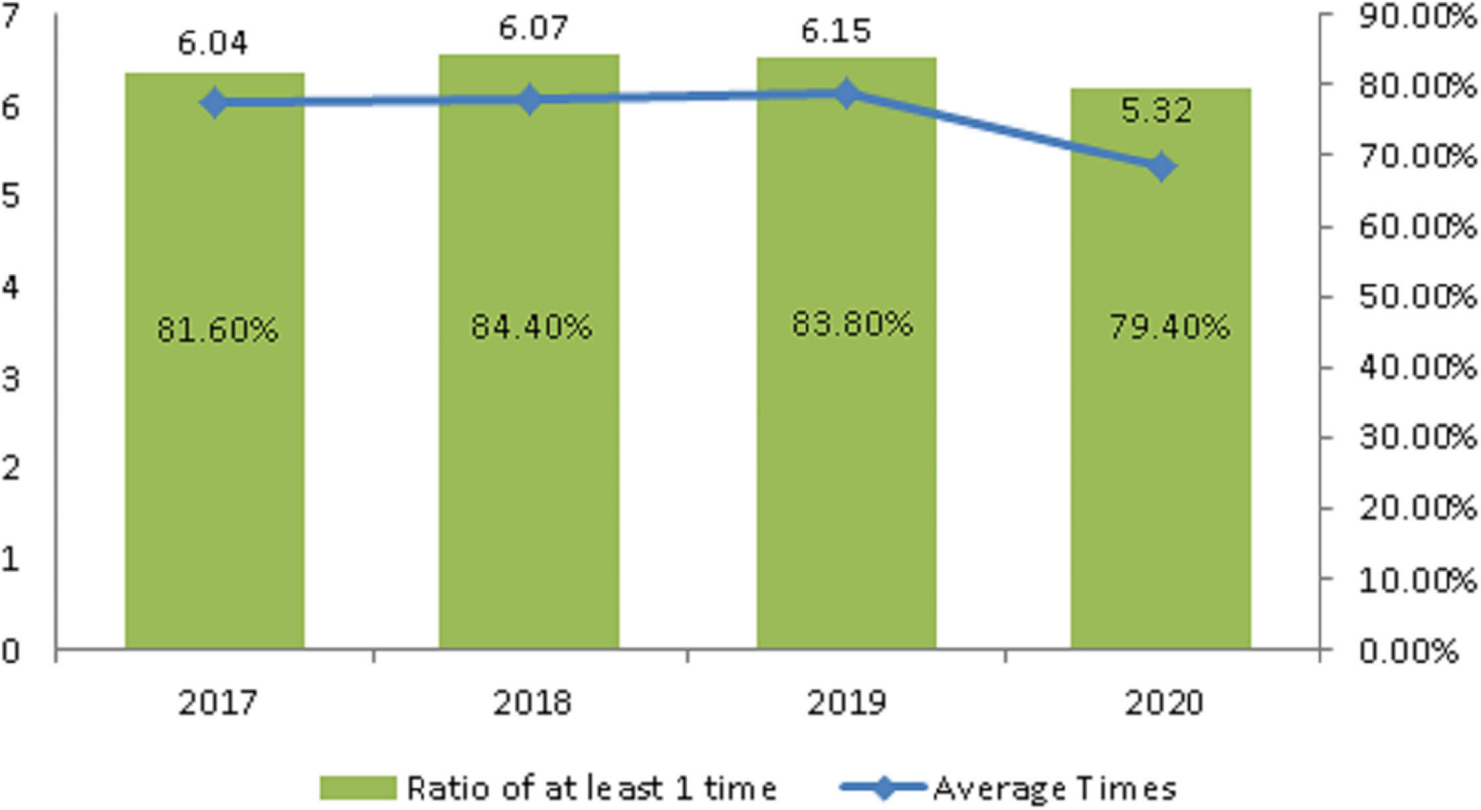
The trend of tourism frequency.

The average number of domestic trips for older people aged 65 and older was 6.04, 6.07, and 6.15 in 2017, 2018, and 2019, respectively (Figure 1). In the 3 years before the outbreak, the average number was 6.09. In 2020, after the outbreak, the number was 5.32, a decrease of 0.77 (Table 2).

**Table 2:** Frequency of tourism.

**Year**	**Average times**	**Rate of at least 1 trip**
2017	6.04	81.60%
2018	6.07	84.40%
2019	6.15	83.80%
2020	5.32	79.40%
Before the pandemic (3-year average)	6.09	83.27%
Post-pandemic change	−0.77	−3.87%

The rate at which people took at least one trip was 81.6, 84.4, 83.8% in 2017, 2018, and 2019, respectively (Figure 1). The average rate in the 3 years before the outbreak was 83.27%. In 2020, after the outbreak, it was 79.4%, a decrease of 3.87% (Table 2).

It can be seen that the average number of trips and the rate of tourism both declined as a result of the pandemic.

### Days of Tourism

**Figure 2:**
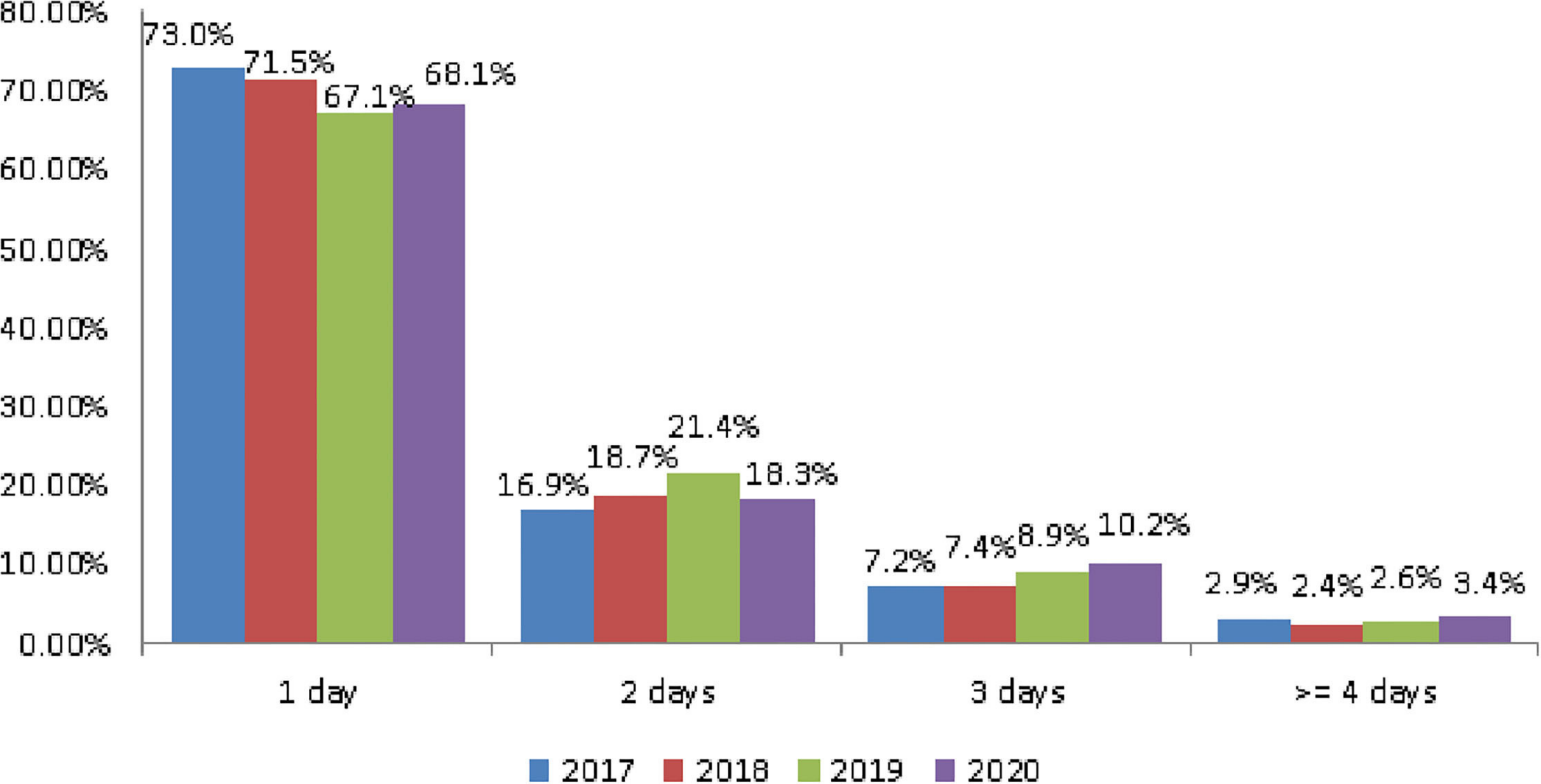
The trend of tourism days.

The tourism day of the older people 65 ages and older was mainly 1 day. The rate was 73, 71.5, and 67.1% in 2017, 2018, and 2019, respectively (Figure 2). The average rate in the 3 years before the outbreak was 70.53%. In 2020, after the outbreak, it was 68.1%, a decrease of 2.43% (Table 3).

**Table 3:** Days of tourism.

**Year**	**1 day**	**2 days**	**3 days**	**≥4 days**
2017	73.00%	16.90%	7.20%	2.90%
2018	71.50%	18.70%	7.40%	2.40%
2019	67.10%	21.40%	8.90%	2.60%
2020	68.10%	18.30%	10.20%	3.40%
Before the pandemic (3-years average)	70.53%	19.00%	7.83%	2.63%
Post-pandemic change	−2.43%	−0.70%	2.37%	0.77%

However, the rate of older people tourism 3 days was 10.2% after the outbreak, an increase of 2.37% compared with 7.83% before the outbreak (Table 3).

It can be seen that as a result of the pandemic, although the 1-day travel rate decreased, the 3-day travel rate increased, and the rate of increase and decrease is similar.

### Purpose of Tourism

**Figure 3:**
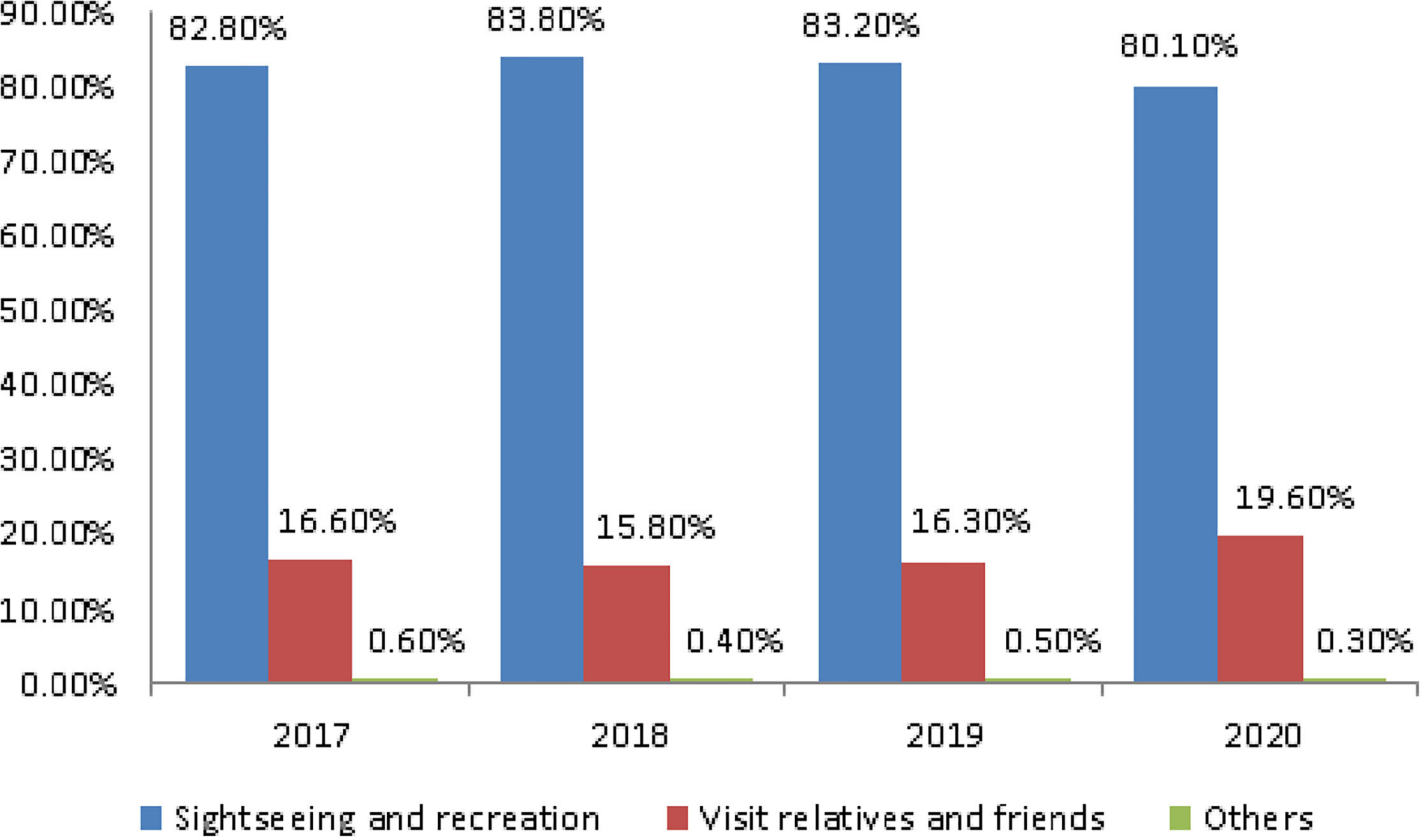
The trend of tourism purpose.

The purpose of tourism for people aged 65 and older was mainly sightseeing and recreation. The rate was 82.8, 83.8, and 83.2% in 2017, 2018, and 2019, respectively (Figure 3). The average rate in the 3 years before the outbreak was 83.27%. In 2020 after the outbreak, it was 80.1%, a decrease of 3.17% (Table 4).

**Table 4:** The purpose of tourism.

**Year**	**Sightseeing and recreation**	**Visiting relatives and friends**	**Other**
2017	82.80%	16.60%	0.60%
2018	83.80%	15.80%	0.40%
2019	83.20%	16.30%	0.50%
2020	80.10%	19.60%	0.30%
Before the pandemic (3-year average)	83.27%	16.23%	0.50%
Post-pandemic change	−3.17%	3.37%	−0.20%

However, after the outbreak, the rate of visiting relatives and friends was 19.6%, compared with 16.23% before the pandemic, an increase of 3.37% (Table 4).

It can be seen that although the rate of sightseeing and recreation decreased owing to the impact of the pandemic, the rate of visiting relatives and friends increased, and the rate of increase and decrease is similar.

### Reasons for Choosing a Tourist Attraction

**Figure 4:**
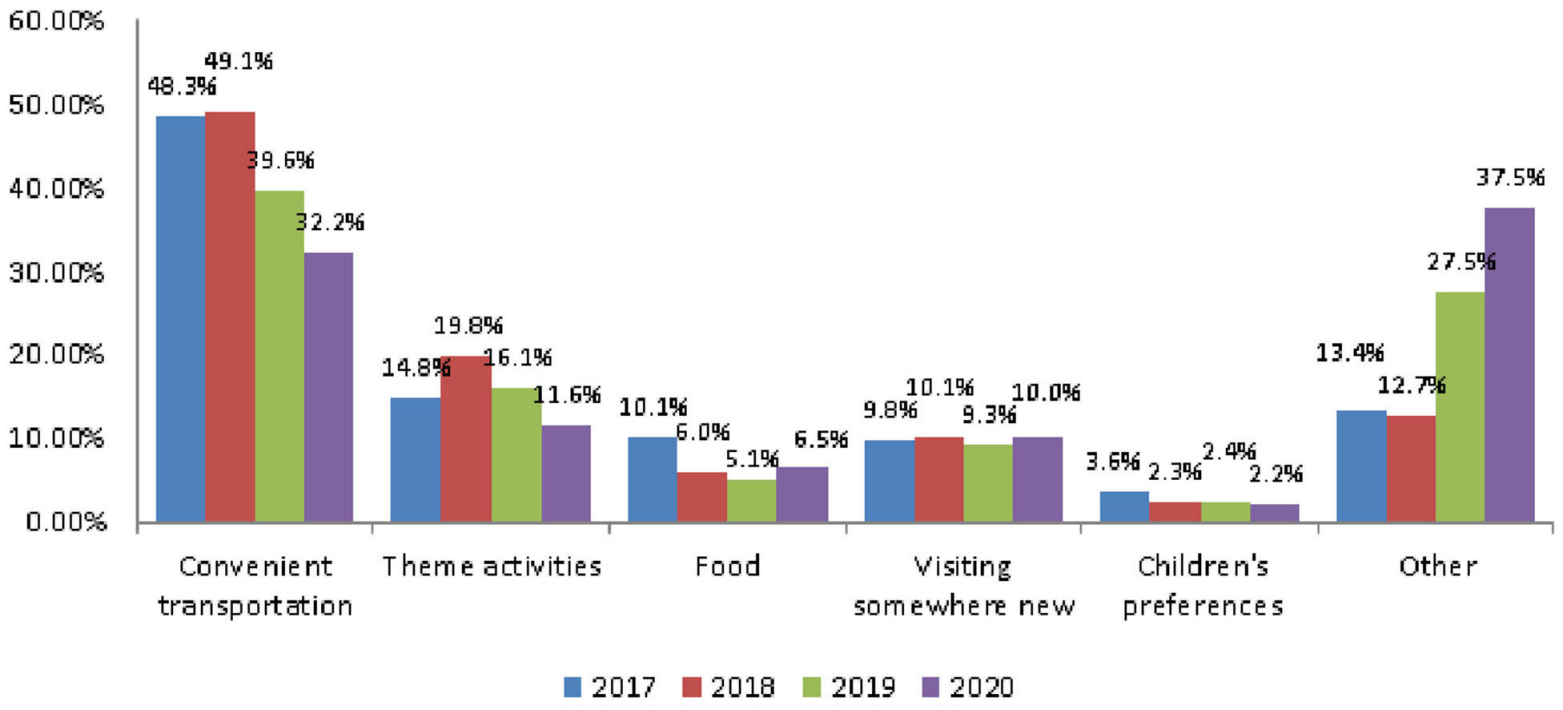
The trend of reasons for choosing a tourist attraction.

The reasons people aged 65 and older chose tourist attractions included convenient transportation, themed activities, food, visiting somewhere new, and their children's preferences. Among them, convenient transportation was the main reason, accounting for 48.3, 49.1, and 39.2% in 2017, 2018, and 2019, respectively (Figure 4). The average rate in the 3 years before the outbreak was 45.67%. In 2020, after the outbreak, it was 32.2%, a decrease of 13.47%. However, after the outbreak of the pandemic, leisure and health care accounted for 16.5%, and fewer crowds for 6% (Table 5).

**Table 5:** The reasons for choosing a tourist attraction.

**Year**	**Convenient transportation**	**Themed activities**	**Food**	**Visiting somewhere new**	**Children's preferences**	**Other**
2017	48.30%	14.80%	10.10%	9.80%	3.60%	13.40%
2018	49.10%	19.80%	6.00%	10.10%	2.30%	12.70%
2019	39.60%	16.10%	5.10%	9.30%	2.40%	27.50%
2020	32.20%	11.60%	6.50%	10.00%	2.20%	37.50%
Before the pandemic (3-years average)	45.67%	16.90%	7.07%	9.73%	2.77%	17.87%
Post-pandemic change	−13.47%	−5.30%	−0.57%	0.27%	−0.57%	19.63%

“*2020 other” includes the “leisure and health care” and “fewer crowds” factors, 16.50 and 6.00%, respectively*.

It can be seen that the pandemic caused the importance of transportation convenience as a factor to fall, but the rate of fewer crowds to increase. Because leisure and health care only began to be represented in the survey data in 2019, it was 16.6%, similar to that in 2020.

### Reasons for not Participating in Tourism

**Figure 5:**
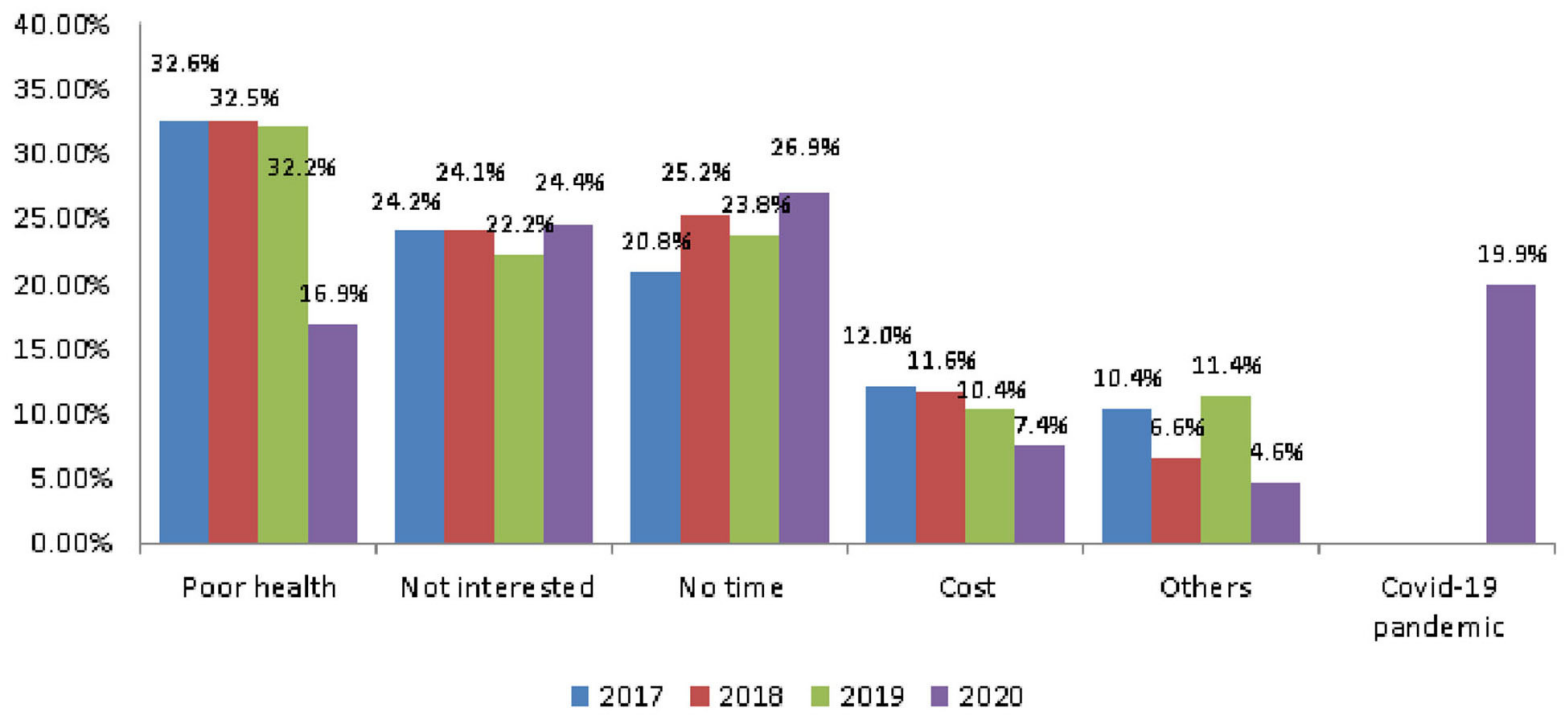
The trend of reasons for not participating in tourism.

The reasons that people aged 65 and older did not participate in tourism included poor health, not being interested, not having time, and cost. Among them, poor health was the main reason; the rates were 32.6, 32.5, and 32.2% in 2017, 2018, and 2019, respectively (Figure 5). The average rate in the 3 years before the outbreak was 32.43%. After the outbreak, it was 16.9% in 2020, a decrease of 15.53%. However, the COVID-19 pandemic factor was 19.9% in 2020 (Table 6).

**Table 6:** Reasons for not participating in tourism.

**Year**	**Poor health**	**Not interested**	**No time**	**Cost**	**Others**	**COVID-19 pandemic**
2017	32.60%	24.20%	20.80%	12.00%	10.40%	NA
2018	32.50%	24.10%	25.20%	11.60%	6.60%	NA
2019	32.20%	22.20%	23.80%	10.40%	11.40%	NA
2020	16.90%	24.40%	26.90%	7.40%	4.60%	19.90%
Before the pandemic (3-year average)	32.43%	23.50%	23.27%	11.33%	9.47%	NA
Post-pandemic change	−15.53%	0.90%	3.63%	−3.93%	−4.87%	19.90%

The rate of poor health care as a factor decreased as a result of the pandemic. However, the increase in the rate of COVID-19 as a factor could also be regarded as a consideration of the overall health factor, and the total rate increased compared with that before the pandemic.

### Tourism Expenses

**Figure 6:**
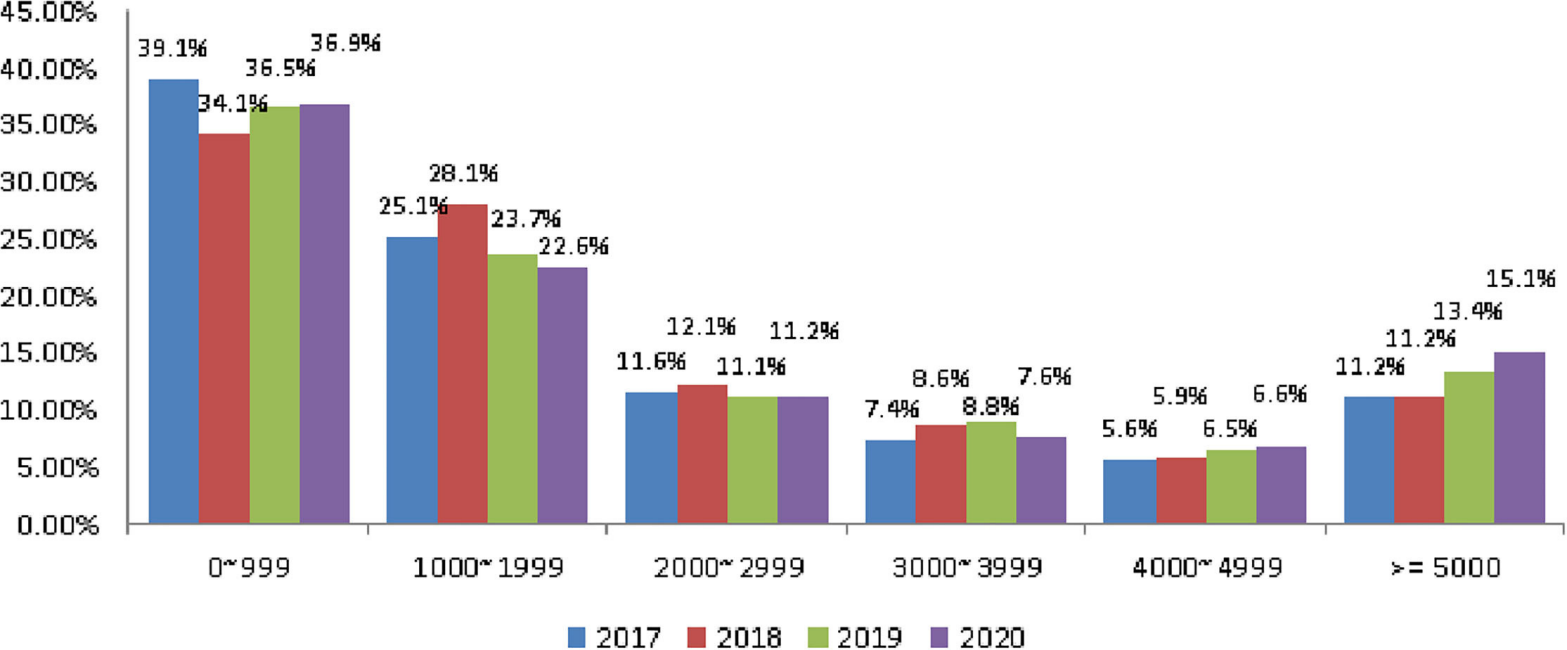
The trend of tourism expenses. Currency unit is NT$. Exchange rate of NT$ to US$ is ~0.035.

People aged 65 and older generally spend <NT$2,000 per tourist trip. The rate of spending <NT$1000 was the highest, which was 39.10, 34.1, and 36.5% in 2017, 2018, and 2019, respectively (Figure 6). The average rate in the 3 years before the outbreak was 36.57%. In 2020 after the outbreak, it was 36.9%, an increase of 0.33%. However, after the pandemic, the rate from NT$1,000 to NT$1,999 was 22.6% compared with 25.63% before the pandemic, a decrease of 3.03%, and an increase of 3.17% for above NT$5,000 (Table 7).

**Table 7:** Tourism expenses.

**Year**	**0–999**	**1000–1999**	**2000–2999**	**3000–3999**	**4000–4999**	**≥5000**
2017	39.10%	25.10%	11.60%	7.40%	5.60%	11.20%
2018	34.10%	28.10%	12.10%	8.60%	5.90%	11.20%
2019	36.50%	23.70%	11.10%	8.80%	6.50%	13.40%
2020	36.90%	22.60%	11.20%	7.60%	6.60%	15.10%
Before the pandemic (3-year average)	36.57%	25.63%	11.60%	8.27%	6.00%	11.93%
Post-pandemic change	0.33%	−3.03%	−0.40%	−0.67%	0.60%	3.17%

*Currency unit is NT$. Exchange rate of NT$ to US$ is ~0.035*.

**Figure 7:**
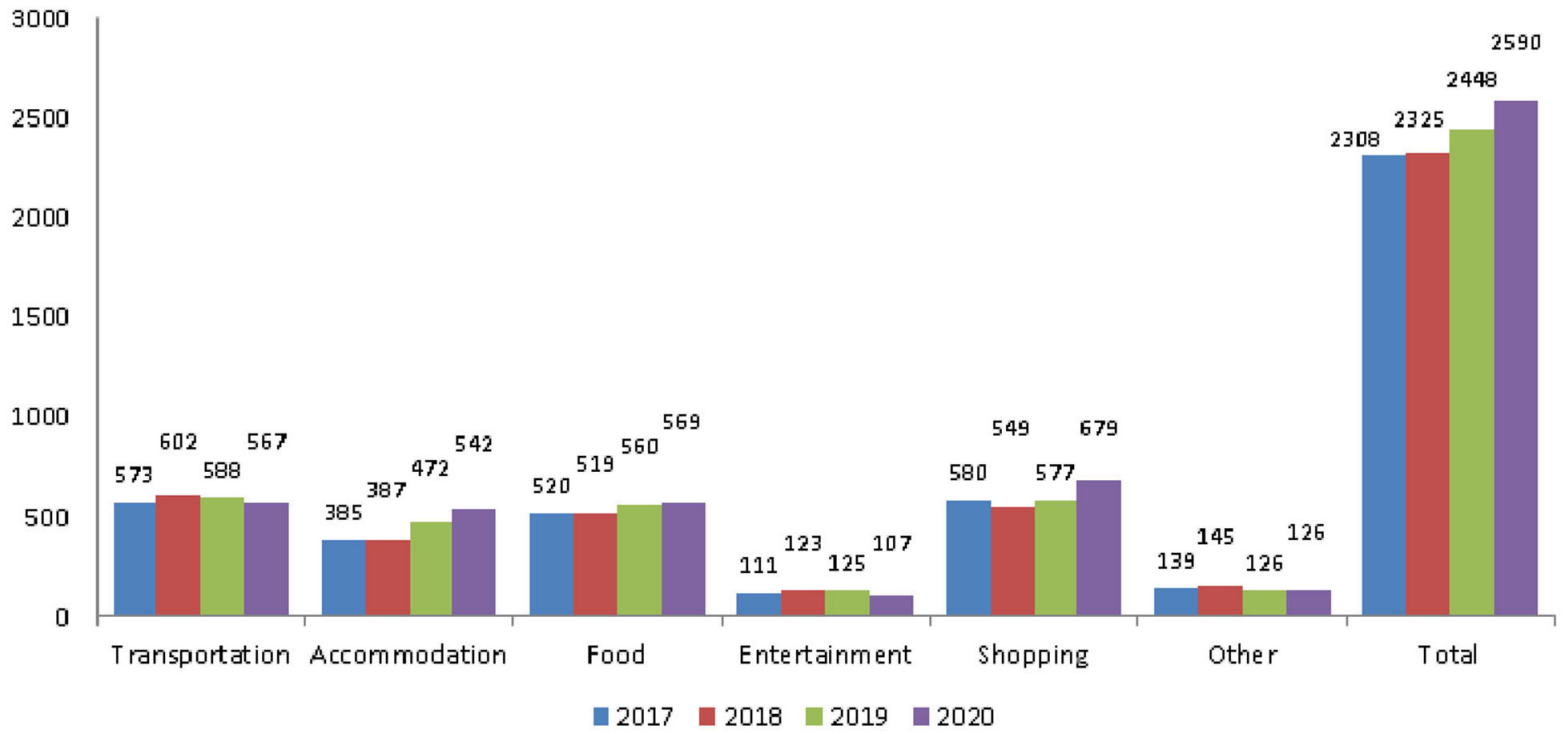
The trend of expenses items. Currency unit is NT$. Exchange rate of NT$ to US$ is ~0.035.

Expenses included transportation, accommodation, food, entertainment, and shopping. The average total amounts were NT$2,308, NT$2,325, and NT$2,448 in 2017, 2018, and 2019, respectively (Figure 7). The average amount in the 3 years before the outbreak of COVID-19 was NT$2,360.33. In 2020, after the outbreak, it was NT$2,590, an increase of NT$229.67 (Table 8). Total travel expenses were not affected by the pandemic and still increased gradually.

**Table 8:** Expenses items.

**Year**	**Transportation**	**Accommodation**	**Food**	**Entertainment**	**Shopping**	**Other**	**Total**
**2017**	573	385	520	111	580	139	2,308
**2018**	602	387	519	123	549	145	2,325
**2019**	588	472	560	125	577	126	2,448
**2020**	567	542	569	107	679	126	2,590
**Before the pandemic (3-year average)**	587.67	414.67	533.00	119.67	568.67	136.67	2,360.33
**Post-pandemic change**	−20.67	127.33	36.00	−12.67	110.33	−10.67	229.67

**Figure 8:**
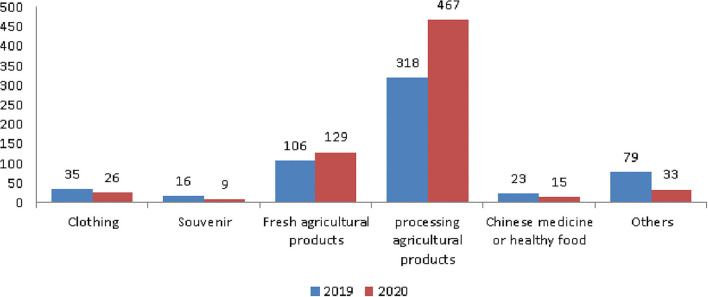
The trend of shopping items. Currency unit is NT$. Exchange rate of NT$ to US$ is ~0.035.

The details of shopping expenses included clothing, souvenirs, fresh agricultural products, processing agricultural products, and traditional Chinese medicine or health food. Among them, processing agricultural products was the main expense (Figure 8). In 2019, it was NT$318. In 2020, it was NT$467, an increase of NT$149 (Table 9). Since shopping items were only included in the survey from 2019, only 2019 and 2020 data exist.

**Table 9:** Shopping items.

**Year**	**Clothing**	**Souvenirs**	**Fresh agricultural**	**processing**	**Chinese medicine**	**Other**
			**products**	**agricultural products**	**or healthy food**	
2019	35	16	106	318	23	79
2020	26	9	129	467	15	33
Post-pandemic changes	−9	−7	23	149	−8	−46

*Currency unit is NT$. Exchange rate of NT$ to US$ is ~0.035*.

### PCA Analysis

**Figure 9:**
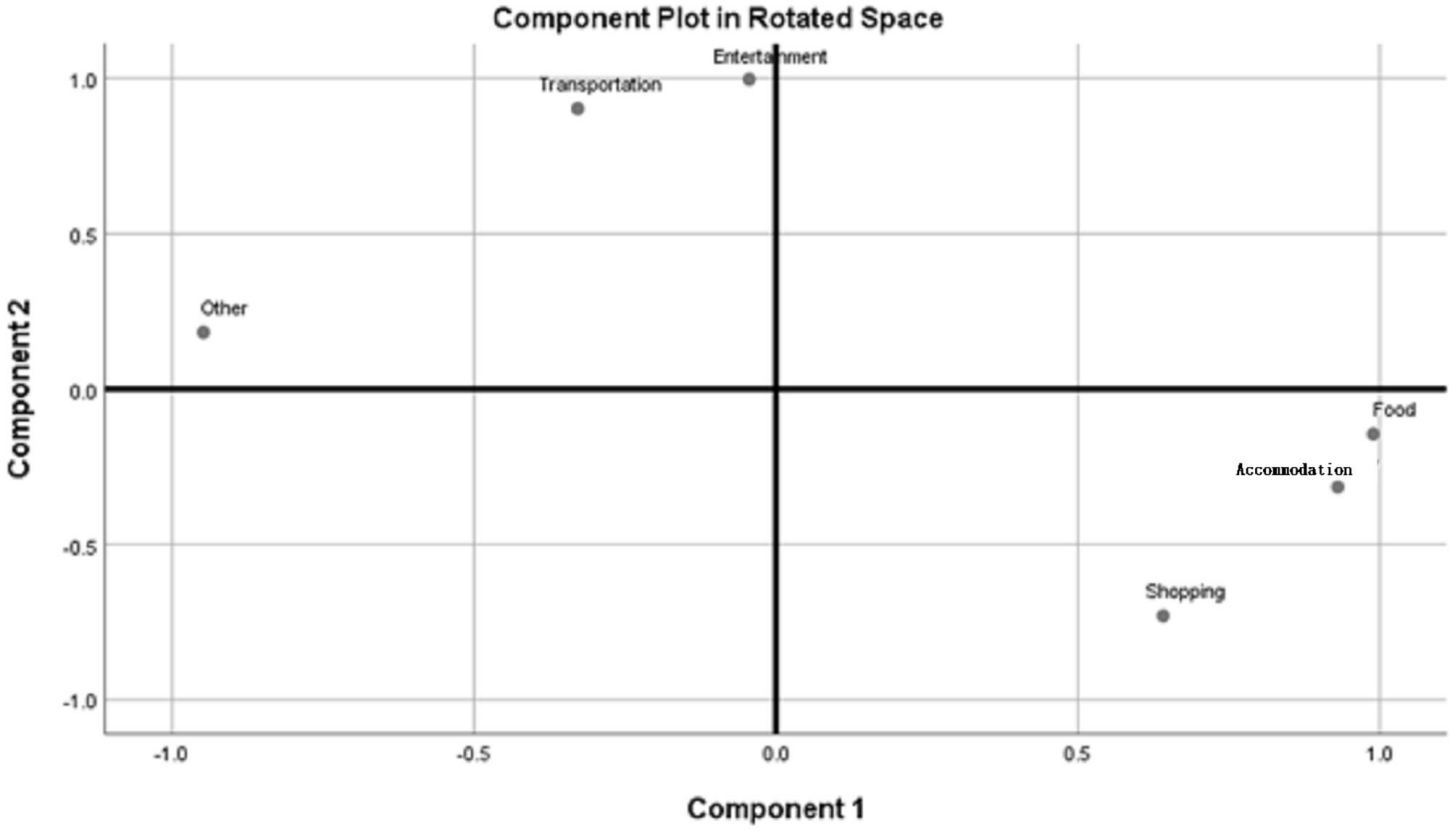
The PCA analysis results of tourism expenses.

There are many tourism costs and related variables. To further understand the relationships between the items on which older people tourists spent money, a PCA analysis was applied to the amount spent on each item. PCA was performed on older people tourists' expenses from 2017 to 2020, and two main components were extracted. The PCA results are shown in Table 10 and Figure 9.

**Table 10:** PCA total variance explained.

**Component**	**Initial eigenvalues**			**Extraction sums of squared loadings**			**Rotation sums of squared loadings**		
	**Total**	**% of variance**	**Cumulative %**	**Total**	**% of variance**	**Cumulative %**	**Total**	**% of variance**	**Cumulative %**
1	4.349	72.49	72.49	4.349	72.49	72.49	3.265	54.414	54.414
2	1.41	23.495	95.985	1.41	23.495	95.985	2.494	41.572	95.985
3	0.241	4.015	100						
4	6.08E-18	1.01E-16	100						
5	−1.43E-16	−2.38E-15	100						
6	−4.70E-16	−7.83E-15	100						

The first component was food, accommodation, shopping, and other expenses. The factor loadings were 0.989, 0.931, 0.641, and −0.948, respectively. The second component was entertainment and transportation expenses. The factor loadings were 0.997 and 0.902, respectively. There was a high and significantly positive relationship between food, accommodation, and shopping expenses. Older people tourists who spent more on food also spent more on accommodation and shopping, but had lower spending on other expenses. On the other hand, older people tourists who spent more on entertainment also spent more on transportation.

## Discussion and Suggestions

From the above statistical and analysis results, we provide several suggestions for relevant government departments and industry operators.

1. Before the COVID-19 pandemic, the average number of tours was 5.32 and the rate of participating in tours was 79.4%. Both showed a decline: of 0.77 number and 3.87%, respectively (Figure 1 and Table 2). Older people's psychological distress, fear, and related behavior was affected by the COVID-19 pandemic (15–19). Therefore, if the pandemic can be controlled, it will help to increase the frequency of travel. The pandemic control factors are mainly based on the vaccination rate and pandemic prevention measures (28). The vaccination rate in Taiwan had reached 80% by the end of January 2022 (29). The effectiveness of the current government's anti-pandemic measures, mean that its confirmed rate and mortality rate are lower than the global average, which is also worth affirming (7, 8).
2. The number of days older people tourists engaged in tourism, regardless of the pandemic situation, was predominantly 1 day, accounting for about 70% of tourism by this group (Table 3). Therefore, relevant industries could launch 1-day travel itineraries to meet the demand. However, there is still a lot of space for operators to develop tourism for longer than 1 day (Figure 2).
3. The purpose of tourism for the older people is still mainly sightseeing and recreation, regardless of the pandemic situation. The rate was 82.8, 83.8, 83.2, and 80.1% in 2017, 2018, 2019 and 2020, respectively (Figure 3). Therefore, it is recommended that the relevant operators launch sightseeing and recreational itineraries to meet the demand.
4. The main reason older people tourists choose tourist attractions is about 40% for the convenience of transportation before the pandemic (Figure 4). After the pandemic, it dropped to ~30%, the factor of fewer crowds accounted for ~6%, and the factor of leisure and health care also reached ~16% (Table 5). Therefore, the government and the industry should fully plan the transportation network of tourist attractions to provide greater convenience, and improve leisure and health care facilities to attract tourists. Healthcare providers should consider strategies for improving preventive behaviors among the older people to help ease their worries and fears concerning COVID-19 (17).
5. The reasons the older people did not participate in tourism were mainly influenced by health factors before the pandemic. The rate was 32.6, 32.5, and 32.2% in 2017, 2018, and 2019, respectively (Figure 5). In addition to health factors, there are also COVID-19 pandemic factors after the pandemic. The COVID-19 pandemic factor was 19.9% in 2020 (Table 6). Especially recently, some countries have been advocating coexistence with the pandemic and have been relaxing pandemic prevention control (30). Therefore, we suggest that the government and the industry should improve and properly plan health and medical care in travel itinerary, so that they can travel with peace of mind and more healthily. Healthcare providers should consider strategies for improving preventive behaviors among the older people to help ease their worries and fears concerning COVID-19 (17).
6. Tourism spending by the older people has increased each year and has not been affected by the COVID-19 pandemic. The average total amounts was NT$2,308, NT$2,325, NT$2,448, and NT$2,590 in 2017, 2018, 2019, and 2020, respectively (Figure 7). Through PCA analysis, we established that the first components were food, accommodation, shopping, and other expenses. The factor loadings were 0.989, 0.931, 0.641, and −0.948, respectively. The second components were entertainment and transportation expenses. The factor loadings were 0.997 and 0.902, respectively (Figure 9). If the industry planned the relevant consumption mix it could achieve the maximum benefit. The shopping items surveyed since 2019 show that processed agricultural products is the most important expenditure, and it also helps businesses plan related products (Table 9).

## Conclusion

The older population in Taiwan reached ~3.95 million at the end of January 2022, accounting for around 16.9% of the country's total population. As the fertility rate declines and life expectancy increases, the older population will continue to increase toward a super-aged society. Therefore, the tourism demand of the older people will gradually increase and receive attention. At present, the COVID-19 pandemic is not completely under due to variants of the virus. This article explores how older people tourism have been affected by this major international public health event since early 2020. This study compares the differences and changes in the behavior of older people tourism before and after the pandemic through relevant statistical analysis such as quantitative and PCA methods. The study results found the frequency of tourism declined: the number was 5.32, a decrease of 0.77, and instances of people undertaking at least 1 tourist trip decreased by 3.87% after the pandemic. The duration of tourism day was mainly 1 day; at 68.1%, it decreased by 2.43% after the pandemic. The purpose of tourism was mainly sightseeing and recreation; it was 80.1%, a decrease of 3.17% after the pandemic. The convenient transportation is the main reason for choosing the attractions; it was 32.2%, a decrease of 13.47% after the pandemic. The reasons of not participate tourism; in COVID-19 pandemic factor was 19.9%. The total travel expenses was NT$2,590, an increase of NT$229.67, was not affected by the pandemic. We found that the frequency of travel, the number of days, the purpose, the reasons for choosing the attractions, and the reasons for not participating in the tour did change as a result of the impact of the pandemic. However, older people tourists were spending more year by year. Through PCA analysis, we examined the relationships between the items on which older people tourists spent money. The first component was food, accommodation, shopping and other expenses. The factor loadings were 0.989, 0.931, 0.641, and −0.948, respectively. The second component was entertainment and transportation expenses. The factor loadings were 0.997 and 0.902, respectively. Through PCA analysis, we established that it could help the industry to plan the relevant consumption mix to achieve maximum benefit. Finally, the paper puts forward relevant discussions and suggestions on the research results. If the pandemic could be controlled, it would help to increase the frequency of travel. The pandemic control factors are mainly based on the vaccination rate and pandemic prevention measures. The number of days spent on tourism by the older people, regardless of the pandemic situation, was still predominantly 1 day, accounting for about 70%. Therefore, we suggest that the tourism industry could launch 1-day travel itineraries to meet the demand. The purpose of tourism for older people is still mainly sightseeing and recreation; so we recommend that the relevant operators launch sightseeing and recreational itineraries to meet the demand. Convenient transportation is the main reason for choosing the attractions, so the government and the industry should fully plan the transportation network of tourist attractions to promote convenience, and should improve leisure and health care facilities to attract tourists, and make tourism for the older people more healthy and sustainable. In particular, the consideration of health factors is the main reason older people do not participate in tourism. In addition, the health status of the older people is not as good as that of young and middle-aged people, and they are more prone to sudden illness or injury. Therefore, the government and the tourism industry must properly plan the relevant health and medical care in travel itineraries to ensure their safety and health.

Whether the COVID-19 pandemic can be completely controlled in the future is not yet clear. The research in this paper includes analysis before and after the pandemic, it will help relevant units and operators to respond and consider different situations. The proportion of the older population in most countries in the world is also gradually increasing. However, at present, there are few studies on the impact of major public health events, such as COVID-19, on older people tourism. Therefore, this article could also contribute to the research on tourism related to the older people in various countries around the world.

## Data Availability Statement

The original contributions presented in the study are included in the article/supplementary material, further inquiries can be directed to the corresponding author.

## Author Contributions

C-TC designs the study and drafted the manuscript.

## Conflict of Interest

The author declares that the research was conducted in the absence of any commercial or financial relationships that could be construed as a potential conflict of interest.

## Publisher's Note

All claims expressed in this article are solely those of the authors and do not necessarily represent those of their affiliated organizations, or those of the publisher, the editors and the reviewers. Any product that may be evaluated in this article, or claim that may be made by its manufacturer, is not guaranteed or endorsed by the publisher.
